# Evidence for Muscle Cell-Based Mechanisms of Enhanced Performance in Stretch-Shortening Cycle in Skeletal Muscle

**DOI:** 10.3389/fphys.2020.609553

**Published:** 2021-01-08

**Authors:** Atsuki Fukutani, Tadao Isaka, Walter Herzog

**Affiliations:** ^1^Faculty of Sport and Health Science, Ritsumeikan University, Kusatsu, Japan; ^2^Department of Physiology and Pharmacology, Karolinska Institutet, Solna, Sweden; ^3^Faculty of Kinesiology, The University of Calgary, Calgary, AB, Canada

**Keywords:** pre-activation, cross-bridge theory, residual force enhancement, titin, elastic energy storage, eccentric muscle action, human performance, skeletal muscle

## Abstract

Force attained during concentric contraction (active shortening) is transiently enhanced following eccentric contraction (active stretch) in skeletal muscle. This phenomenon is called stretch-shortening cycle (SSC) effect. Since many human movements contain combinations of eccentric and concentric contractions, a better understanding of the mechanisms underlying the SSC effect would be useful for improving physical performance, optimizing human movement efficiency, and providing an understanding of fundamental mechanism of muscle force control. Currently, the most common mechanisms proposed for the SSC effect are (i) stretch-reflex activation and (ii) storage of energy in tendons. However, abundant SSC effects have been observed in single fiber preparations where stretch-reflex activation is eliminated and storage of energy in tendons is minimal at best. Therefore, it seems prudent to hypothesize that factor(s) other than stretch-reflex activation and energy storage in tendons contribute to the SSC effect. In this brief review, we focus on possible candidate mechanisms for the SSC effect, that is, pre-activation, cross-bridge kinetics, and residual force enhancement (RFE) obtained in experimental preparations that exclude/control the influence of stretch-reflex activation and energy storage in tendons. Recent evidence supports the contribution of these factors to the mechanism of SSCs, and suggests that the extent of their contribution varies depending on the contractile conditions. Evidence for and against alternative mechanisms are introduced and discussed, and unresolved problems are mentioned for inspiring future studies in this field of research.

## Introduction

**Figure 1:**
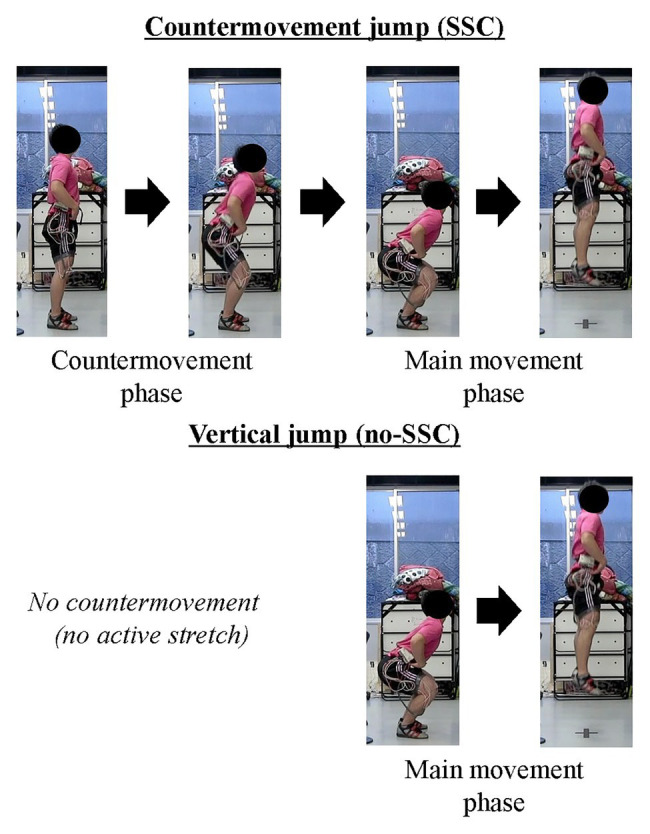
Schematic example of stretch-shortening cycles (SSCs). The upper panel shows the countermovement jump (SSC) and the lower panel shows the squat jump (no SSC). Note that, the countermovement jump includes active stretching phase.

When we attempt to jump as high as possible, we naturally make a countermovement (i.e., eccentric contraction or active stretch) before the main movement (i.e., concentric contraction or active shortening; Figure 1). This is because we empirically know that a countermovement improves the subsequent jump performance. Actively stretching a muscle prior to the muscle producing positive work/power in a shortening contraction is called a stretch-shortening cycle (SSC; Bosco et al., 1982; Komi, 2000). SSCs in skeletal muscle are frequently observed in sport activities and are considered a crucial component for optimal performance, thus, elucidating the mechanisms of the SSC effect has been a central question in sport science for decades. There are many studies on the SSC effect and almost as many proposals for mechanisms underlying the SSC effect. The most frequently discussed mechanisms for the SSC effect are an increase in neural activation in SSCs compared to a pure shortening contraction through stretch-reflex activation (Nichols and Houk, 1973; Dietz et al., 1979) and the storage and subsequent release of energy in series elastic components of the muscle, primarily the free tendon (Kubo et al., 2000; Finni et al., 2001; Kawakami et al., 2002). However, we found a substantial SSC effect in preparations, such as skinned fibers, that do not contain a stretch-reflex activation component and have minimal/negligible possibility for storing energy in tendinous components (Fukutani et al., 2017a). Therefore, it seems prudent to hypothesize that the SSC effect can be produced by mechanisms other than stretch-reflex activation and storage/release of elastic energy, and furthermore, it appears quite possible that the SSC effect is caused by molecular events in the contractile machinery of the muscle itself; that is the actin-myosin-titin complex of sarcomeres. Here, we introduce possible candidate mechanisms for the SSC effect other than stretch-reflex activation and storage/release of elastic energy. This choice should not be interpreted that stretch-reflex activation and energy storage/release do not play a role in the SSC effect in human/animal movement. Rather, we would like to point out that other mechanism might, and likely will contribute to the SSC effect, and in fact may be the primary reasons for the SSC effect. The possible candidate mechanisms we will introduce and discuss are: (i) pre-activation, (ii) cross-bridge kinetics, and (iii) residual force enhancement (RFE). These candidate mechanisms were chosen primarily because of recent evidence as to their importance.

## Pre-Activation

**Figure 2:**
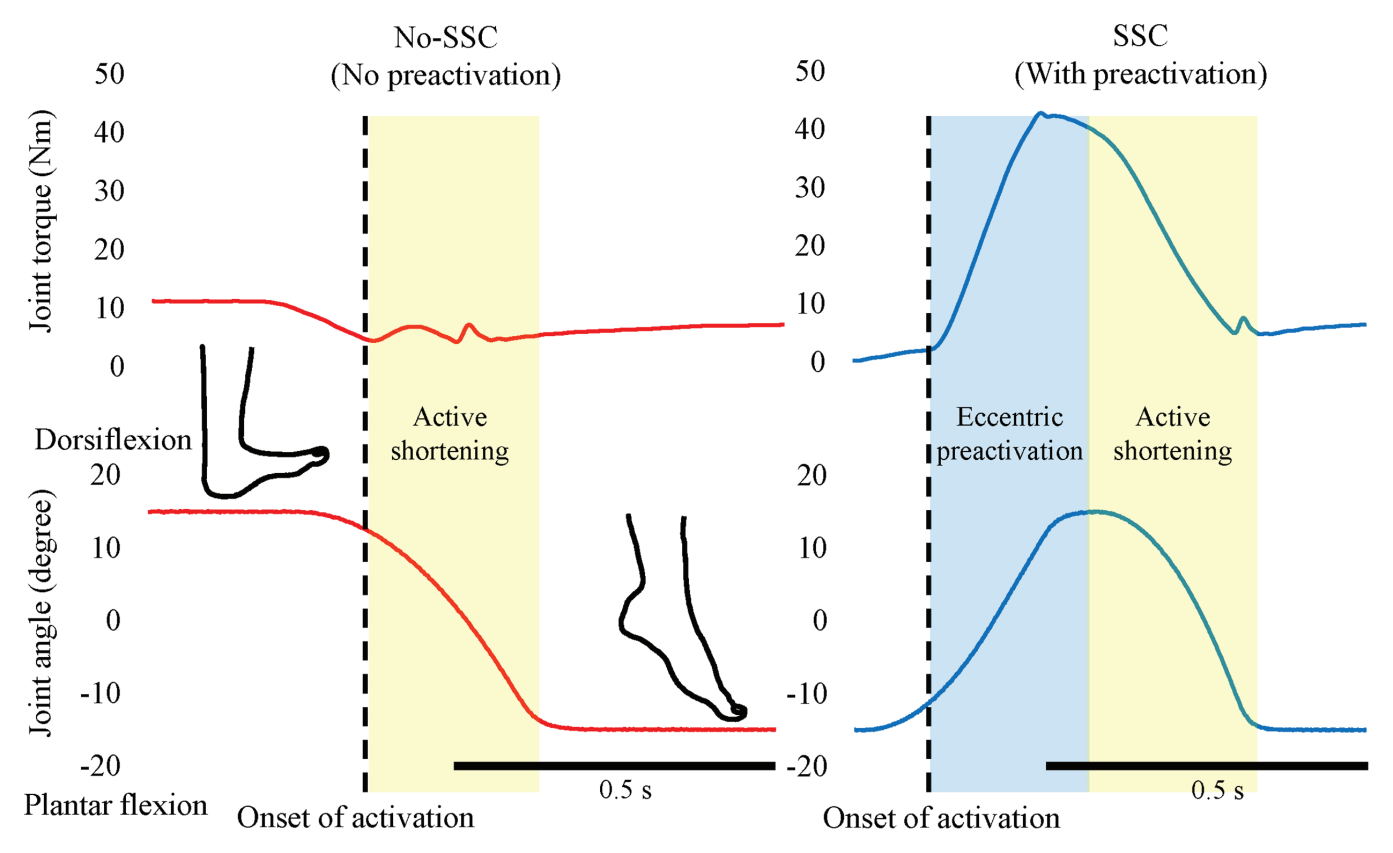
The effect of pre-activation. The left panel shows the No-SSC (no pre-activation) condition and the right panel shows the SSC (with pre-activation condition). The muscle force (joint torque) increases after the onset of activation (dotted lines). Note that, the muscle force increases during the active shortening phase in the no-SSC while continuously decreases in the SSC condition. This difference indicates that the muscle force is not fully developed in the early phase of active shortening in the No-SSC condition. Original figure made from authors’ published data (Reproduced with permission from Fukutani et al., 2015a).

In SSCs, active muscle shortening is preceded by active muscle stretching. Therefore, a muscle is already activated at the onset of active shortening (Figure 2, right panel). In a purely concentric (muscle shortening) contraction, a muscle may not be activated prior to the onset of active shortening (Figure 2, left panel) or may be activated to a smaller degree than during a SSC. For example, in a countermovement vertical jump, the leg extensor muscles are highly activated to stop and reverse the downward movement of the body, while in a squat jump, starting from a stationary position, the leg extensor muscles are activated merely to produce the required joint moments for holding the body stationary, or if the squat jump is executed from a relaxed position (sitting on a chair, for example), no leg extensor activation is required (Figure 1). Since activation in a purely shortening contraction needs to increase from zero or a small value, when initiating the concentric movement, it has been found that active force, especially in the early phase of the shortening phase, is much smaller in a pure shortening movement compared to the corresponding force in a SSC. This pre-activation effect has been widely found in human experiments and simulations (Svantesson et al., 1994; Bobbert et al., 1996; Bobbert and Casius, 2005; Fukutani et al., 2015a, 2016). If pre-activation plays a role in the SSC effect, one might reasonably assume that the difference between pre-activation vs. no pre-activation is great when the concentric muscle phase is short (either because the shortening distance is small or the speed of shortening is high) since the time available to develop the muscle force is limited. Indeed, when shortening given amplitude at different speeds (30 or 150°/s of human plantar flexion) after active muscle stretching, the SSC effect was greater for the fast than the slow shortening speed (Fukutani et al., 2015b).

As mentioned in the previous paragraph, it is widely acknowledged that pre-activation contributes to the SSC effect. However, the notion of pre-activation is not well-defined in the literature, which might make the following discussion ambiguous. Here, we briefly discuss the influence of pre-activation on the SSC effect. For example, the force at the onset of shortening is greater when pre-activation is combined with eccentric rather than isometric muscle action. If pre-activation was the primary contributor to the SSC effect, then the contractile conditions during pre-activation phase should not matter. Therefore, pre-activation cannot explain the difference in force at the onset of shortening, and thus, we do not consider this difference as a pre-activation effect. However, a difference in force at the onset of shortening may be a key factor for elucidating the mechanisms underlying the SSC effect. In the following, we would like to introduce possible mechanisms that are independent of pre-activation: these are the cross-bridge kinetics and RFE.

## Cross-Bridge Kinetics

**Figure 3:**
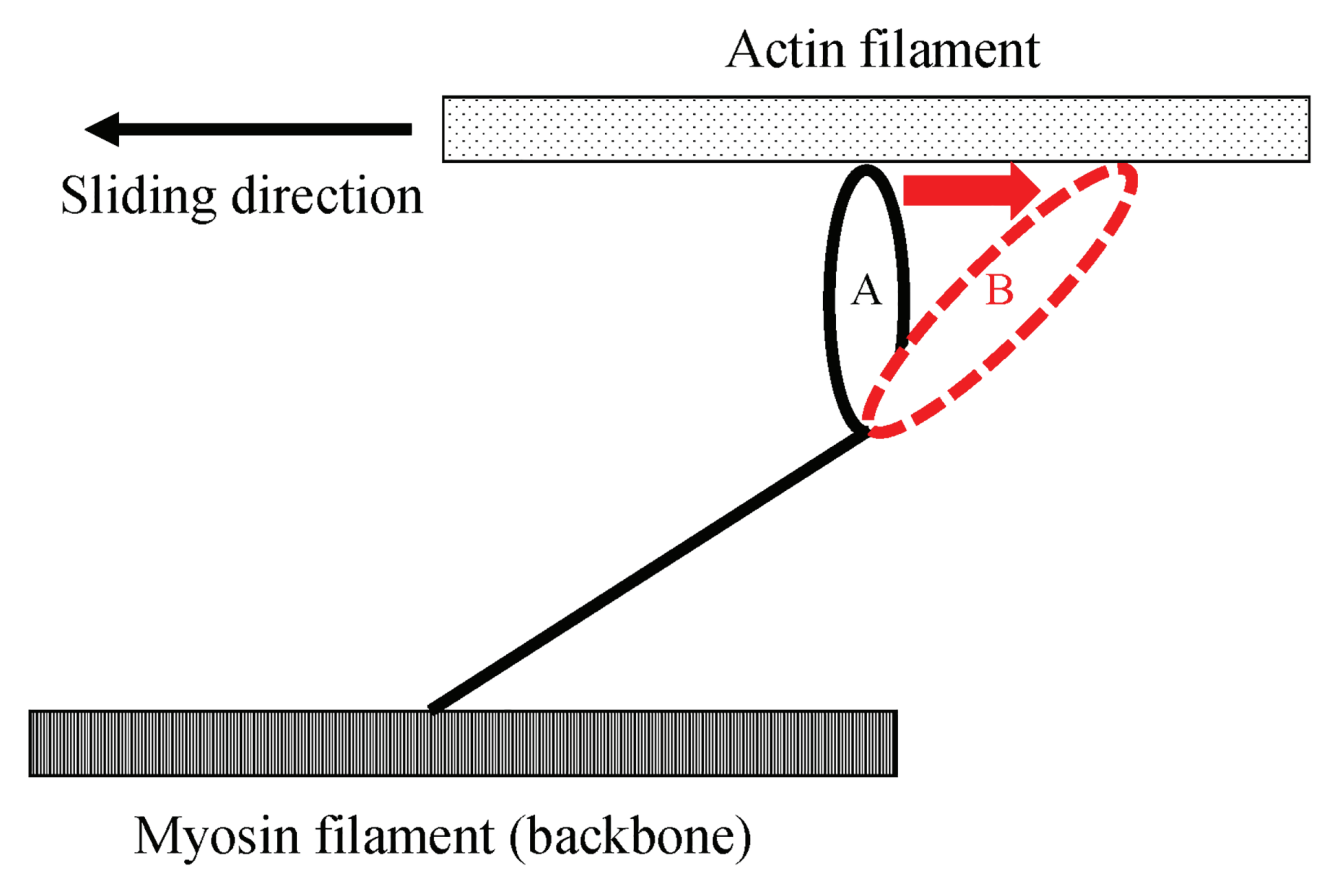
Schematic explanation of the elongated attached cross-bridges by an active stretch. Once muscles are actively elongated to the opposite direction relative to the sliding direction, attached cross bridges are deformed from A to B. Due to this deformation, the elastic energy is stored in the elongated attached cross-bridges.

**Figure 4:**
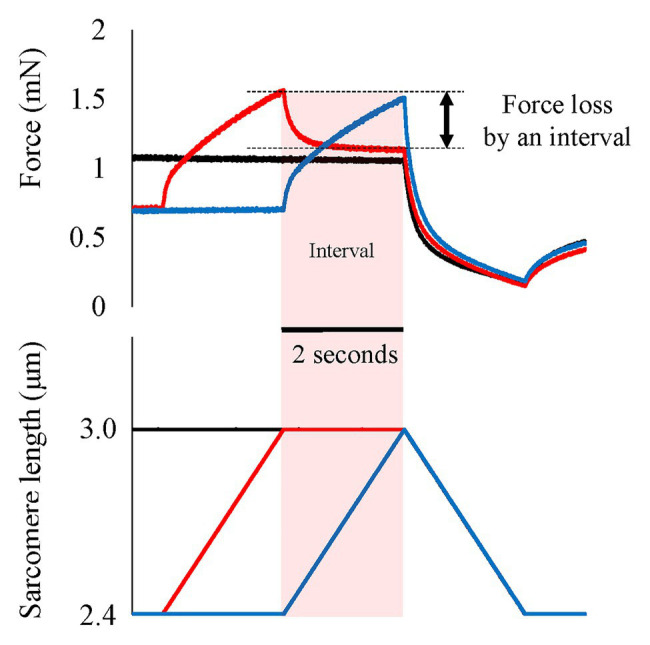
Force and length responses when an interval is provided between active stretch and active shortening. Black line indicates the pure shortening condition (isometric-active shortening). Blue line indicates the normal SSC condition (isometric-active stretch-active shortening). Red line indicates the SSC with interval (isometric-active stretch-isometric-active shortening). In the SSC with interval condition, the force quickly and substantially decreases immediately after the end of active stretch. This force loss would be derived from the detachment of elongated attached cross-bridges. Original figure made from authors’ published data (Reproduced with permission from Fukutani et al., 2017a).

One of the possible candidates for the SSC effect is the cross-bridge kinetics. It is well-known and generally acknowledged that the force at the onset of active shortening is greater after eccentric pre-activation than after isometric pre-activation. This greater force in eccentric vs. concentric contraction is well-explained by the cross-bridge theory and associated force-velocity relationship (Hill, 1938) of skeletal muscles for these two conditions, although the exact molecular mechanisms are still debated (Huxley, 1957; Lombardi and Piazzesi, 1990; Piazzesi et al., 2007). During an active stretch, attached cross-bridges are forcibly elongated, leading to an increase in force per cross-bridge and the storage of elastic energy (Flitney and Hirst 1978a,b; Lombardi and Piazzesi, 1990; Figure 3). These attached cross-bridges have the potential for more mechanical work during a subsequent active shortening compared to “unstretched” cross-bridges in a purely isometric contraction (Bosco and Komi, 1979). A good way to examine cross-bridge states in SSCs is to directly visualize the deformation of the attached cross-bridges in real time. However, it is difficult to do that because of the small cross-bridge movements (less than 10 nm; Kaya and Higuchi, 2013) in a short time (few ms; Capitanio et al., 2006). A less elegant, but simple and practical way to measure the contribution of cross-bridges to the SSC effect is to introduce pauses of varying duration between the stretch and shortening phases of SSCs. If a pause of sufficient duration is provided, attached cross-bridges will detach from actin, and the elastic energy stored in the cross-bridge disappears, thereby eliminating any cross-bridge effect due to active muscle stretching. Indeed, when providing a 1 s (or greater interval) between the stretch and shortening phases of SSCs, the SSC effect has been found to be attenuated. This finding has been consistently observed in human movement performance (vertical jump in Bosco et al., 1981, plantar flexion in Fukutani et al., 2017b) and in skinned single fiber experiments (psoas in Fukutani et al., 2017a; Figure 4). These results fit nicely the textbook explanation that *“If a concentric muscle action does not occur immediately following the eccentric action, the stored energy dissipates and is lost as heat”* (Coburn and Malek, 2011). If this description is correct, the effect of tendon elongation, i.e., storing elastic energy in tendons, might be reconsidered. If the elastic energy stored in the tendon is a primary contributor to the SSC effect, this stored elastic energy should be dissipated quickly once an interval is provided. However, tendons are almost elastic elements with little viscous behavior and a hysteresis of typically less than 10% (Ker, 1981; Bennett et al., 1986). Based on these characteristics, the elastic energy stored in tendons would not dissipate substantially even after providing a short interval (1–2 s) between the stretch and shortening phase of SSCs, suggesting that tendon elongation, and associated storage of potential energy, cannot explain the empirically well-supported characteristic that the SSC effect disappears when a short pause is given between the stretch and shortening phase of SSCs. In contrast, storage of elastic energy in cross-bridges would be fully dissipated in a 1 s pause, thereby supporting the notion of cross-bridge kinetics as a main contributor to the SSC effect.

**Figure 5:**
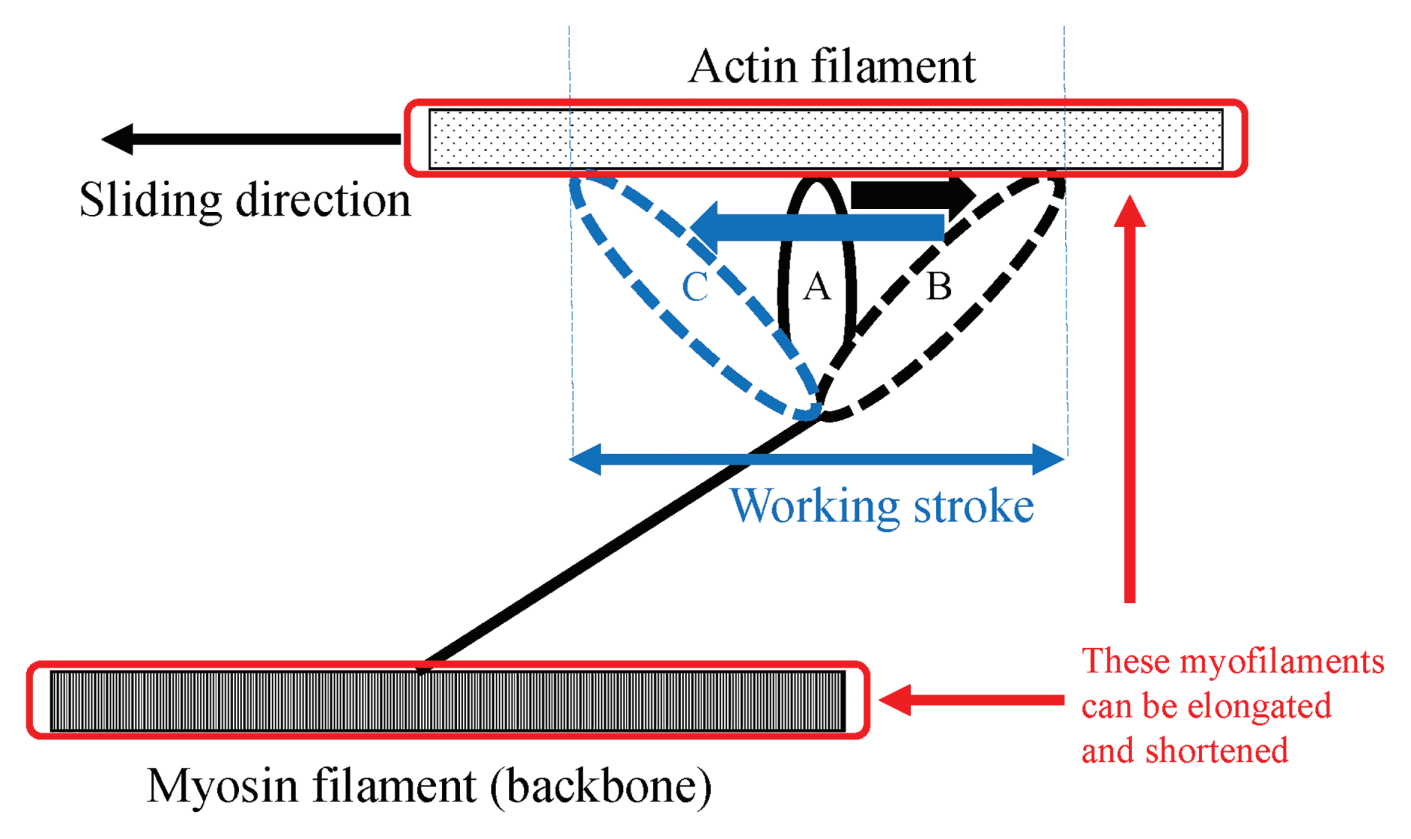
Schematic explanation of the shortening of sarcomere after an active stretch. Due to an active stretch, attached cross-bridges are elongated from the position A to the position B. Then, the elongated attached cross-bridges moves from the position B to the position C during the subsequent active shortening. This movement is called the working stroke. In addition to this deformation of the attached cross-bridges, myosin, and action filaments can be elongated and shortened because these filaments also have compliance. Therefore, the shortening/lengthening of sarcomere is composed of not only attached cross-bridges but also the myofilaments.

**Figure 6:**
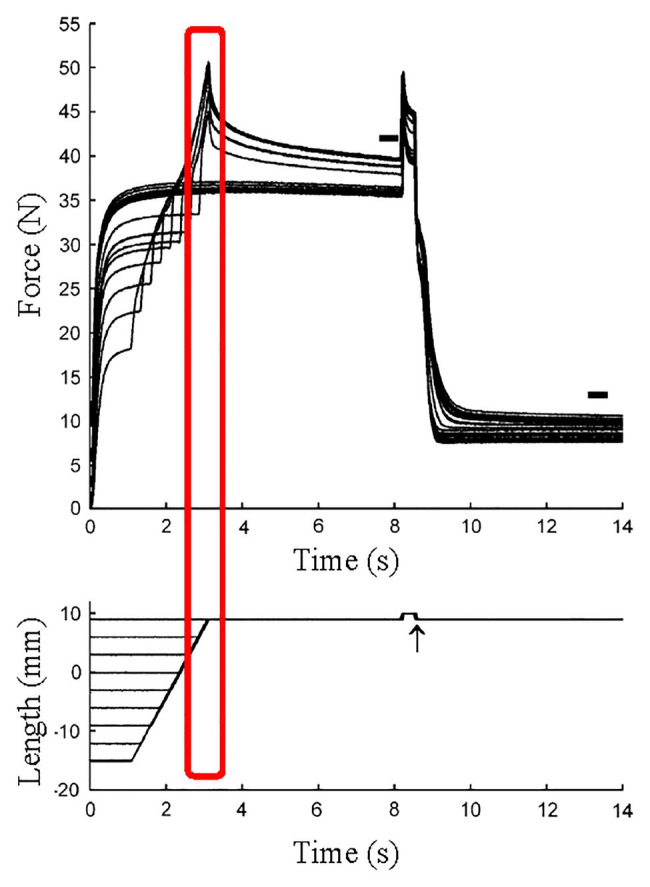
Force and length responses in different stretch magnitude with the identical stretch velocity and the identical final muscle length. Although the stretch velocity (force-velocity relationship) and the final muscle length (force-length relationship) were the same among conditions, the force at the end of active stretch was different among conditions. This result indicates that factor(s) other than cross-bridges contributed to the difference in force (Reproduced with permission from Bullimore et al., 2007).

If elongated attached cross-bridges indeed contribute to the SSC effect, their contribution should be limited to the early phase of the active shortening, as it is generally assumed that the elastic energy stored in attached cross-bridges is dissipated once detachment has occurred. Thus, the time and/or shortening distance available for cross-bridges to contribute to the SSC effect is limited, and corresponds at most to one cross-bridge cycle. Cross-bridge detachment occurs in a matter of ms in a mammalian skeletal muscle (e.g., 2–20 ms in rat soleus and mouse gastrocnemius, Capitanio et al., 2006) and is restricted, on average to a half sarcomere shortening of about 5 nm (Kaya and Higuchi, 2013), possibly 10 nm considering the elongation of attached cross bridges by active stretching (Figure 5). Therefore, if a half sarcomere shortens by more than about 10 nm, cross-bridge based elastic energy storage cannot contribute further to the SSC effect (Huxley, 1957; from position B to position C in Figure 6). In addition, compliance of sarcomere is not only derived from attached cross-bridges, but also from the actin and myosin filaments (Huxley et al., 1994; Wakabayashi et al., 1994; Colombini et al., 2005; Nocella et al., 2011). Based on these findings, 50% of sarcomere elongation can be derived from the elongation of myofilaments (Figure 6, red squares), that is, another 10 nm/half sarcomere elongation can be expected, and a part of this elongation can be converted into sarcomere shortening during a cross-bridge cycle. In total, one would expect at most 20 nm (2% of half sarcomere length) of shortening in a single cross-bridge cycle. Thus, if cross-bridge elongation was the sole factor for the SSC effect, the SSC effect would be limited to the initial 2% of sarcomere length shortening. Findings in human muscle and single skinned fiber preparations showed that most of the SSC effect disappears in the initial shortening phase of SSCs (Fukutani et al., 2015a,b, 2016, 2017a,b), thereby supporting the idea that the cross-bridge kinetics might be a significant contributor to the SSC effect.

It has been argued, based on stiffness measurements that the number of attached cross-bridges is greater in an eccentric compared to an isometric or concentric contraction (Lombardi and Piazzesi, 1990; Brunello et al., 2007; Fusi et al., 2010). Therefore, not only cross-bridges contain more elastic potential energy after stretch, but also there would be more of them attached to actin just prior to shortening in a SSC compared to shortening preceded by an isometric contraction. Furthermore, due to the greater eccentric force compared to the isometric force, one would expect more myosin cross-bridges to be in the so-called “ON” state, a state that allows for quick re-attachment of detached cross-bridges and is favored by high muscle force, rather than the “OFF” state, which is characterized by a slow re-attachment rate, and is favored by low muscle force (Fusi et al., 2016). An increased number of attached cross-bridges and a great percentage of cross-bridges in the “ON” state following an eccentric muscle action might be important contributors to the SSC effect. At present, these two phenomena have been examined only for isolated isometric and/or isolated eccentric conditions (Lombardi and Piazzesi, 1990; Brunello et al., 2007; Fusi et al., 2010), but have not been examined in the context of SSCs, i.e., after active stretching. Therefore, what role they may play and what their contribution to the SSC effect may be await scientific investigation.

When muscles are stretched quickly with small stretching magnitude (about 1% of fiber length within 1 ms), force after stretch (transient force) increases, a phenomenon referred to as stretch activation (Campbell and Chandra, 2006). Because of its properties, stretch activation may contribute to the SSC effect. Stretch activation has been found to be of substantial magnitude, and thus is thought to be of functional relevance, in insect flight muscles and cardiac muscles, while stretch activation seems to be small/negligible in vertebrate skeletal muscles (Perz-Edwards et al., 2011). For these reasons, we assume that the influence of stretch activation on the SSC effect in mammalian skeletal muscles is small at best.

## Residual Force Enhancement

In addition to the cross-bridge kinetics, evidence seem to suggest that there are other factors that contribute to the SSC effect. For example, in preparations in which stretch-reflex activation and tendon compliance were eliminated, some SSC effect was still present even after introducing a long pause between the stretch and shortening phases of SSCs (Cavagna et al., 1968; Fukutani et al., 2017a,b). This suggests a mechanism that persists beyond the time of cross-bridge cycling whose effect is still present seconds after the stretch phase has finished. RFE has been shown to be present following eccentric muscle action and persist for a long time; minutes in single skinned fiber and myofibril preparations, and thus, offers itself naturally as a candidate mechanism for the long-lasting SSC effect.

Residual force enhancement is a property of skeletal muscle characterized by an increase in the steady-state isometric force after an active stretch compared to the corresponding purely isometric steady-state force attained at the same muscle length and activation level (Abbott and Aubert, 1952; Edman et al., 1978). Although RFE has been associated primarily with the development of sarcomere length non-uniformity (Morgan, 1990, 1994), evidence have accumulated over the past 30 years that likely, RFE is associated with the engagement of a passive structural element (Noble, 1992; Forcinito et al., 1998), and there is accumulating evidence that this structural element is the filamentous protein titin (Labeit et al., 2003; Joumaa et al., 2007, 2008; Leonard and Herzog, 2010; DuVall et al., 2013, 2017). A proposed mechanism of titin-induced RFE is a shortening of titin’s free spring length by titin’s proximal segment binding to actin (Herzog, 2018). The actin filament has several potential binding sites for titin (Linke et al., 1997; Kulke et al., 2001; Bianco et al., 2007). If the proximal segment of titin (proximal Ig domain) is bound to actin, the proximal segment of titin cannot be elongated during active stretch. To compensate for this, the distal segment of titin is elongated more at a given stretch magnitude. This leads to the increased force at a given sarcomere length, that is, RFE (Herzog, 2018). In this review article, we tentatively consider that RFE is primally induced by titin, not sarcomere length non-uniformity (Morgan, 1990, 1994). The most striking reason is that recent studies which visualized the sarcomere length changes before and after active stretch found that although sarcomere length non-uniformity was observed after active stretch, a similar non-uniformity in sarcomere length was also observed before active stretch and during isometric reference contractions (Joumaa et al., 2008; Johnston et al., 2016). The other reasons are discussed in detail in our recent review article (Fukutani and Herzog, 2019a). Based on these points, we will assume here, for the sake of argument, that titin is the primary mechanism for RFE although direct and unequivocal evidence for titin’s contribution to RFE is still lacking. RFE is typically defined as the increase in “steady-state isometric force” following active muscle stretching compared to the corresponding force for a purely isometric contraction (Herzog and Leonard, 2002). However, it is well accepted that RFE is established during the active stretch phase of muscles (e.g., Edman et al., 1982; Bullimore et al., 2007), and not only steady-state isometric contraction after stretching, and thus we consider all effects of enhanced force during and after stretching as manifestations of RFE. This definition includes increases in force during “active shortening” that is preceded by muscle stretching.

Since RFE is induced by stretching an active muscle, it seems reasonable to speculate that REF is induced during SSCs. Previous studies revealed that the force at the end of a stretch at a given speed and final muscle length increased with increasing stretch magnitude (Schachar et al., 2004; Bullimore et al., 2007; Figure 6). Furthermore, it has been shown that the peak force following stretching to a given length, and associated RFE are highly correlated, suggesting that these results are caused by RFE that builds up and increases with increasing stretch magnitude of muscles (Bullimore et al., 2007). These results cannot be explained within the framework of the cross-bridge theory (Huxley, 1957) or the force-velocity and force length relationships (Hill, 1938; Gordon et al., 1966).

**Figure 7:**
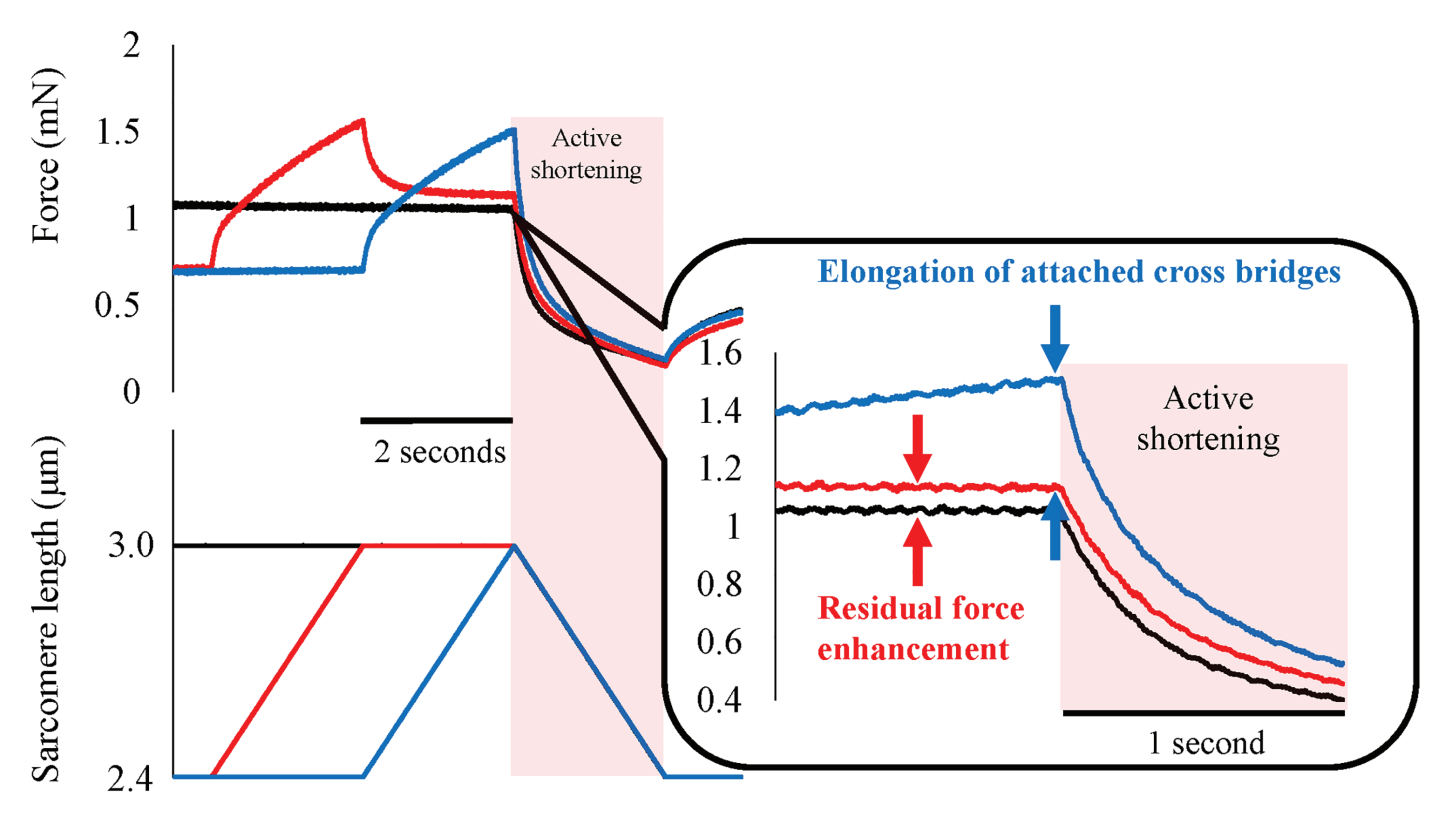
Force and length responses in with and without interval conditions. Black line indicates the pure shortening condition (isometric-active shortening). Blue line indicates the normal SSC condition (isometric-active stretch-active shortening). Red line indicates the SSC with interval (isometric-active stretch-isometric-active shortening). Although the force in the SSC with interval condition decreased after the end of active stretch, the force at the onset of active shortening was still greater compared to the force attained in the pure shortening condition (red arrows). This result indicates the existence of residual force enhancement (RFE; Reproduced with permission from Fukutani et al., 2017a).

In order to determine the possible contribution of RFE to the SSC effect, it is essential to distinguish between the contribution of the RFE and all the other possible sources, such as, the effects of stretched cross-bridges, stretch-reflex activation, and storage/release of energy in tendons and other series elastic elements of muscles. To achieve conditions where only RFE could contribute to the SSC effect, we conducted experiments in skinned fiber preparations where the storage/release of elastic energy is minimal and stretch-reflex activation is fully abolished. Furthermore, by inserting an appropriate pause between the stretch and shortening phase of SSCs, any effect of elongated cross-bridges could also be eliminated. Providing a pause of 2 s still resulted in greater force at the onset of shortening and greater work during shortening for SSC conditions compared to conditions where shortening was not preceded by muscle stretching (Fukutani et al., 2017a; Figure 7, red line vs. black line). This result implies that the SSC effect observed under these conditions is likely caused exclusively by the RFE.

In order to further determine the contribution of RFE to the SSC effect, we conducted a series of further experiments, as follows: First, we conducted SSC experiments with different stretch magnitudes but identical stretch velocities and identical final muscle length. Since the RFE is known to be greater for increasing magnitudes of muscle stretching (Edman et al., 1978; Schachar et al., 2004; Herzog and Leonard, 2005; Bullimore et al., 2007), one would expect the corresponding mechanical work to increase as well in the active shortening phase of such SSCs, which is indeed what we observed (Fukutani and Herzog, 2019b). Second, we also conducted the SSC experiments in which the stretch magnitude and velocity were kept the same, but the testing was performed either on the plateau or the descending limb region of the force-length relationship (Fukutani and Isaka, 2019). RFE for identical stretch conditions is known to be greater on the descending limb compared to the plateau region (Julian and Morgan, 1979; Morgan et al., 2000; Peterson et al., 2004), while the effect of cross-bridges should be smaller on the descending limb than the plateau region because the number of attached cross-bridges is smaller on the descending limb. Therefore, one might expect that the SSC effect is greater for SSC experiments performed on the descending limb than on the plateau region of the force-length relationship if RFE contributes to the SSC effect. As we hypothesized, the magnitude of the RFE was greater on the descending limb than the plateau region (Fukutani and Isaka, 2019).

**Figure 8:**
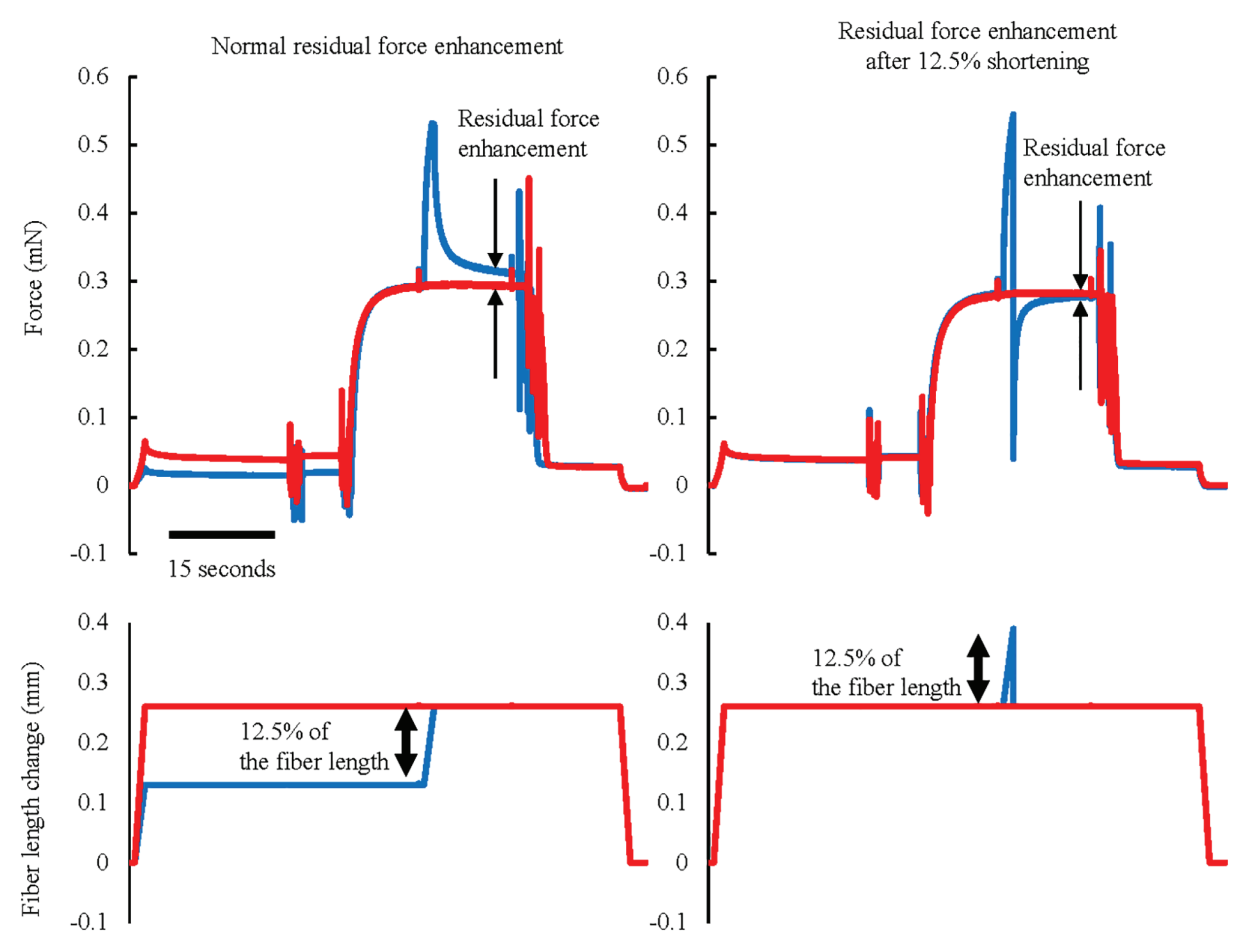
Force and length responses in normal RFE and RFE after 12.5% shortening conditions. Red lines indicate the reference isometric contraction at an average sarcomere length of 3.0 μm and blue lines indicate the RFE condition. RFE was observed by 12.5% of fiber length active stretch but this RFE was substantially attenuated by the same amount of quick shortening performed immediately after the active stretch (Reproduced with permission from Fukutani and Herzog, 2018).

Finally, let us consider the effect of active shortening on RFE. Generally, the properties of RFE have been determined by experiment in which an active stretch preceded an “isometric contraction.” However, there are few natural movements that contain a stretch of an active muscle followed by a long, isometric contraction, In contrast, many natural movements contain SSCs. In SSCs, the active stretch of a muscle is followed by an “active shortening,” rather than an isometric contraction. For human movement analyses, it might be important to know if the active shortening positively or negatively affects the RFE built up in the stretch phase of SSCs. RFE is long-lasting, in excess of 30 s in skinned fibers and myofibrils, where fatigue is not a confounding factor (Joumaa et al., 2008). However, it is unknown if the RFE is also retained during and following active muscle shortening. Herzog et al. (2003) found in cat soleus muscle that passive force enhancement, which is thought to be induced by a change in titin elasticity, was eliminated instantaneously by a passive shortening in a shortening magnitude-dependent manner. This result suggests that the RFE may be attenuated by mechanical shortening. Moreover, we recently conducted an experiment in which an active muscle stretch was followed by a very quick shortening phase (12.5% of fiber length shortening in 0.5 ms; Figure 8). Since active muscle shortening is known to produce force depression (Abbott and Aubert, 1952; Maréchal and Plaghki, 1979), there are two general possibilities for an attenuation of RFE by active shortening; (1) shortening inherently eliminates the RFE or (2) active shortening-induced force depression offsets the stretch-induced RFE. To distinguish between these two possibilities, we performed experiments in which an active muscle stretch was followed by a very quick shortening step of significant magnitude. In these experiments, the force in the quick shortening step decreases quickly because the attached cross-bridges become slack rapidly to a degree where they cannot produce force (Huxley and Simmons, 1971). Force depression increases with increasing muscle force/work in the active shortening phase (Herzog and Leonard, 1997; De Ruiter et al., 1998). Therefore, it is hypothesized that the effect of force depression can be minimized by quick shortening. Since the quick shortening step performed in our experiments was associated with little/negligible force/work, we can test if active shortening reduces the magnitude of RFE in the absence of significant shortening-induced force depression. And indeed, a quick shortening step that was associated with negligible force depression eliminated a great part of the stretch-induced RFE, suggesting that shortening of a muscle itself, in the absence of force depression, reduces the amount of RFE (Figure 8, right panel; Fukutani and Herzog, 2018). In the context of SSCs, this result indicates that the magnitude of the RFE is attenuated by shortening in SSCs. In addition, RFE tends to be smaller at shorter muscle lengths and is said to be negligible for some lengths on the ascending limb of the force-length relationship (Julian and Morgan, 1979; Morgan et al., 2000; Peterson et al., 2004). Therefore, any contribution of RFE to the shortening phase of SSCs would be diminished, possibly negligible for short muscle length on the ascending part of the force-length relationship. These factors should be taken into account when discussing the possible contribution of RFE to the SSC effect.

## Factors Modulating the Contribution on the SSC Effect

If the above three factors contribute to the SSC effect, we can speculate that the contribution of each factor can be modulated dependent on the situations. For example, shortening velocity (duration) should affect the magnitude of SSC effect because the effect of pre-activation should become smaller when the shortening velocity is slow enough to develop the force after initiating the shortening without pre-activation (Fukutani et al., 2015b, 2016).

In addition, stretching velocity would also affect the magnitude of the SSC effect. The reason for the velocity dependence may be associated with the recent observation that high velocities of stretch that resulted in muscle slippage, were associated with a decrease in RFE compared to the RFE obtained for slow and moderate stretch velocities (Fukutani et al., 2019c). Thus, it is likely that the SSC effect is also reduced when the stretch velocity is beyond a critical threshold that causes muscle slippage and a corresponding decrease in RFE.

Moreover, fiber type might affect the SSC effect because of differences in cross-bridge kinetics between fast and slow twitch fibers (Capitanio et al., 2006) and possible differences in titin elasticity (Prado et al., 2005). Recently, we compared the SSC effect between fast (rabbit psoas) and slow (rabbit soleus) twitch fibers and found that the SSC effect was greater in the slow compared to the fast twitch fibers while RFE was the same (Fukutani and Herzog, 2020). These results suggest that the increased SSC effect in the slow fibers is associated with the slower cross-bridge kinetics compared to the fast fibers and is not caused by differences in RFE.

If cross-bridge and RFE (titin) contribute to the SSC effect, the relative contribution of these factors can be identified by changing the cross-bridge kinetics while maintaining titin function or vice versa. This may be achieved, for example with so-called reduced force conditions in which cross-bridge action is submaximal due to submaximal activation, fatigue, chemical inhibition, or genetic modifications. For these conditions, one would expect the contribution of myosin cross-bridges to decrease while the contribution of RFE associated with passive structures, such as titin or myosin binding protein C, might be preserved. We recently confirmed that the magnitude of RFE was preserved in reduced force conditions including those achieved by chemical inhibition of myosin cross-bridges. Thus, it is reasonable to assume that contributions to the SSC effect not produced by cross-bridges, for example titin, become more prominent in reduced force states. This hypothesis has been tested using a myosin inhibitor, blebbistatin, and it was found that the SSC effect was still observed even though the active force was substantially decreased (Tomalka et al., 2020). Similarly, temperature may affect the SSC effect. Differences in force at different temperatures are thought to be caused primarily by the proportion of attached cross-bridge (Wang and Kawai, 2001). If this is indeed the case, one would expect that the SSC effect caused by cross-bridges becomes smaller while that caused by titin does not change with decreasing temperatures. These factors that may affect the SSC effect should be examined systematically in the future.

## Applicability and Limitations

In this review, we addressed the effects of pre-activation, cross-bridge kinetics, and RFE as possible candidates for the SSC effect. However, we would like to emphasize that the laboratory experiments on isolated muscles and fibers may not necessarily reflect what happens in natural SSCs during human movement. For example, in a countermovement jump, the triceps surae muscles are passively elongated in the initial phase and then actively stretched in the latter phase of the countermovement. Although the triceps surae muscles are stretched in this countermovement phase, the corresponding muscle fibers remain isometric or may even shorten when force increases in this phase of the movement (Fukunaga et al., 2001; Kawakami et al., 2002). It is unclear how changes in activation of a muscle (group) during lengthening affect the amount of RFE, and the corresponding contribution of RFE to the shortening phase of SSCs. In most experimental studies, muscles are activated prior to stretching, or, at a minimum, are activated at the onset of stretching (Seiberl et al., 2015; Fortuna et al., 2017; Fukutani et al., 2017b; Fukutani and Herzog, 2018; Hahn and Riedel, 2018), thus producing different contractile conditions than observed in typical human movements. We recently reported that the timing of activation during the stretch-phase of SSCs is crucial in determining the magnitude of the SSC effect, likely because of differences in fascicle mechanics during the countermovement. Specifically, if counter-movements are performed with the muscle pre-activated, fascicle elongation in the stretch phase of SSCs was increased compared to when there was no pre-activation (Fukutani et al., 2019a,b). Pre-activation also led to greater RFE and an augmented SSC effect compared to the no pre-activation conditions (Fukutani et al., 2019a,b). These results suggest that the effect of RFE in physiological movements might be smaller than typically measured in laboratory experiments.

An additional consideration that may affect the SSC effect is the muscle length at which the SSCs occur. In our experiments on single skinned fiber preparations, we typically worked on the descending limb of the force-length relationship where RFE is known to be greater than at shorter (ascending and plateau regions) fiber lengths (Julian and Morgan, 1979; Morgan et al., 2000; Peterson et al., 2004). Muscle excursions in human movement are typically thought to occur on the ascending limb of the force-length relationship (Burkholder and Lieber, 2001) where RFE is known to be small, or even negligible. For example, the human plantar flexor muscles are thought to operate primarily on the ascending limb of the force-length relationship (Kawakami et al., 1998; Maganaris, 2001), and indeed, we failed to find a statistically significant RFE in plantar flexors when the final muscle length was short (plantar flexion of zero degrees), while we observed a significant amount of RFE when the final muscle length was long (15 degrees of dorsiflexion; Fukutani et al., 2017c).

Taken together, these findings discussed above suggest that results obtained in reduced preparations (isolated muscles, single fibers, and single myofibrils) under laboratory conditions may not necessarily reflect the conditions experienced in SSCs that occur during natural human movements. Therefore, to gain better insight into the mechanics and force response of muscles during SSCs that occur in human movement, experiments adopting more physiological contractile and activation conditions should be performed, in parallel with basic experiments in reduced preparations and well-controlled contractile and activation conditions to elucidate the molecular mechanisms underlying the cross-bridge kinetics and/or the role of RFE in SSCs.

## Conclusion

There is accumulating evidence that stretch-reflex activation and storage/release of energy in tendons cannot explain the entirety of the SSC effect. It appears that contractile and structural protein-based mechanisms, which have received little attention to date, may play a significant, possibly a dominant role in producing the SSC. Since protein-based mechanisms likely involve the contractile proteins actin and myosin and the structural protein titin, it may be difficult to elucidate the mechanism underlying the SSC effect using research based on *in vivo* human voluntary muscle testing. The mechanisms involving cross-bridge kinetics and RFE introduced in this brief review will need direct and independent evaluation before they can be fully accepted/rejected. However, it is our hope that some of the initial support for cross-bridge and RFE based mechanisms may inspire research in this exciting area of muscle mechanics, and may lead to a better understanding of SSCs in skeletal muscle.

## Author Contributions

All authors listed have made a substantial, direct and intellectual contribution to the work, and approved it for publication.

### Conflict of Interest

The authors declare that the research was conducted in the absence of any commercial or financial relationships that could be construed as a potential conflict of interest.
